# Supplementary material for: Microbial thermogenesis is dependent on ATP concentrations and the protein kinases ArcB, GlnL, and YccC
Source: PLoS Biol. 2023 Oct 20;21(10):e3002180. doi: 10.1371/journal.pbio.3002180 (PMC10619766; doi:10.1371/journal.pbio.3002180)
Supplement: S1 Note — If a gene was not found, it was searched under its various pseudonyms from MetaCyc. If a gene could not be found, it was not tested. This was the case for 5 transcription factors. Five proteins listed as transcription factors for E. coli K-12 were unable to be found in the Keio Collection under their listed name or any synonyms. These genes were: mazE, mqsA, dicA, birA, and dnaA. This is because these genes are essential to the E. coli K-12 BW25113 strain. (DOCX) [file pbio.3002180.s001.docx]

**S1 Note**

The script scans through a predefined list of target genes and parses through the Keio Collection library to locate the position of the user-defined genes of interest. If a gene was not found, it was searched under its various pseudonyms from MetaCyc. If a gene could not be found, it was not tested. This was the case for five transcription factors. Five proteins listed as transcription factors for *E. coli* K-12 were unable to be found in the Keio Collection under their listed name, or any synonyms. These genes were: *mazE*, *mqsA*, *dicA*, *birA*, and *dnaA*. This is because these genes are essential to the *E. coli* K-12 BW25113 strain.
