# Supplementary material for: Microbial thermogenesis is dependent on ATP concentrations and the protein kinases ArcB, GlnL, and YccC
Source: PLoS Biol. 2023 Oct 20;21(10):e3002180. doi: 10.1371/journal.pbio.3002180 (PMC10619766; doi:10.1371/journal.pbio.3002180)
Supplement: S2 Note — Supply of these nutrients meant, under the assumption that heat generation is due to catabolism of nutrients, that the ideal conditions for maximal heat generation were provided for cells. However, note that in the calorimeter, cells cannot be growing completely aerobically as there is no air flow into the ampoule, and the calorimeter does not allow for shaking, meaning poor aeration of media. Future experimentation will be aimed at understanding how the oxygen concentration changes with time in the ampoule and affects heat output. Additionally, a total of 449 experimental knockout strains were tested, representing 370 unique knockout strains. The number of experimental strains is greater than the unique tested strains due to replicate testing. (DOCX) [file pbio.3002180.s002.docx]

**S2 Note**

The media in this study were chosen to supply the necessary nutrients for core metabolism. Supply of these nutrients meant, under the assumption that heat generation is due to catabolism of nutrients, that the ideal conditions for maximal heat generation were provided for cells. However, note that in the calorimeter, cells cannot be growing completely aerobically as there is no air flow into the ampoule, and the calorimeter does not allow for shaking, meaning poor aeration of media. Future experimentation will be aimed at understanding how the oxygen concentration changes with time in the ampoule and affects heat output. Additionally, a total of 449 experimental knockout strains were tested, representing 370 unique knockout strains. The number of experimental strains is greater than the unique tested strains due to replicate testing.
