# Supplementary material for: Microbial thermogenesis is dependent on ATP concentrations and the protein kinases ArcB, GlnL, and YccC
Source: PLoS Biol. 2023 Oct 20;21(10):e3002180. doi: 10.1371/journal.pbio.3002180 (PMC10619766; doi:10.1371/journal.pbio.3002180)
Supplement: S2 Table — The standard error of the estimates (SEE) of microbial thermogenesis of the sum of the 2 Gaussian equations (2G) and microbial thermogenesis model (MTM) are tabulated. Error from peak calculation takes the value of SEE and divides it by the average peak thermogenesis to understand the variance of the average peak thermogenesis. (DOCX) [file pbio.3002180.s005.docx]

**S2 Table: Standard error of the MTM estimate.** The standard error of the estimates (SEE) of microbial thermogenesis of the sum of the two Gaussian equations (2G) and microbial thermogenesis model (MTM) are tabulated. Error from peak calculation takes the value of SEE and divides it by the average peak thermogenesis to understand the variance of the average peak thermogenesis.

**
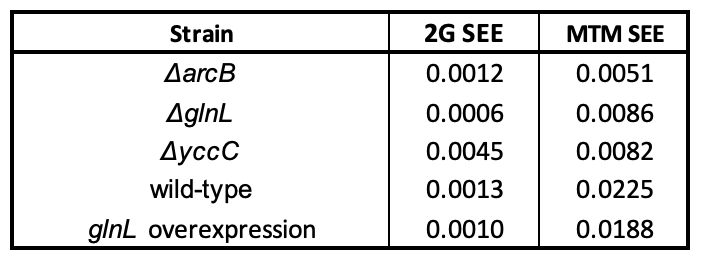
**
