# Supplementary material for: Microbial thermogenesis is dependent on ATP concentrations and the protein kinases ArcB, GlnL, and YccC
Source: PLoS Biol. 2023 Oct 20;21(10):e3002180. doi: 10.1371/journal.pbio.3002180 (PMC10619766; doi:10.1371/journal.pbio.3002180)

**S3 Table: Growth model selection.** A replicate test was used to test for model fit to the data. Comparison between three commonly used models of population growth of cells: Gompertz Growth, Malthusian Growth, and Exponential Plateau. The p-values and rejection of model decisions are shown as calculated.


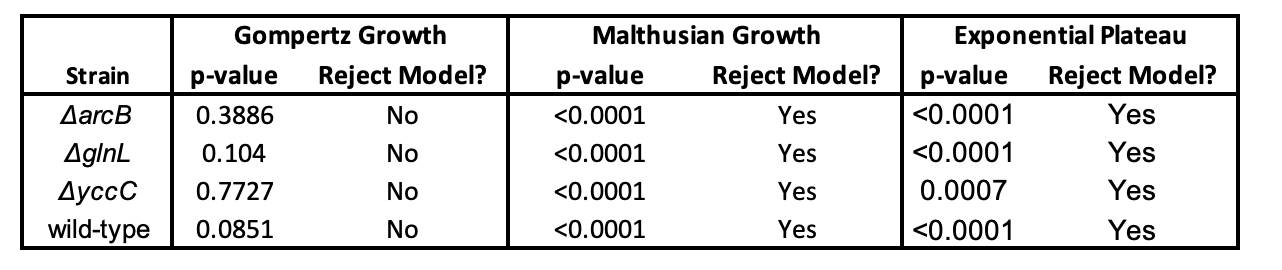

Supplement: S3 Table — A replicate test was used to test for model fit to the data. Comparison between 3 commonly used models of population growth of cells: Gompertz Growth, Malthusian Growth, and Exponential Plateau. The p-values and rejection of model decisions are shown as calculated. (DOCX) [file pbio.3002180.s006.docx]
