# Supplementary material for: Microbial thermogenesis is dependent on ATP concentrations and the protein kinases ArcB, GlnL, and YccC
Source: PLoS Biol. 2023 Oct 20;21(10):e3002180. doi: 10.1371/journal.pbio.3002180 (PMC10619766; doi:10.1371/journal.pbio.3002180)
Supplement: S4 Table — Comparison between 7 different models for microbial heat generation curves. A replicate test was used to test for model fit to the data with p-values less than 0.01 defined as a rejection of the model. Statistical analyses were conducted in GraphPad Prism 9. (DOCX) [file pbio.3002180.s007.docx]

**S4 Table: Thermogenesis model selection.** Comparison between seven different models for microbial heat generation curves. A replicate test was used to test for model fit to the data with p-values less than 0.01 defined as a rejection of the model. Statistical analyses were conducted in GraphPad Prism 9.

**
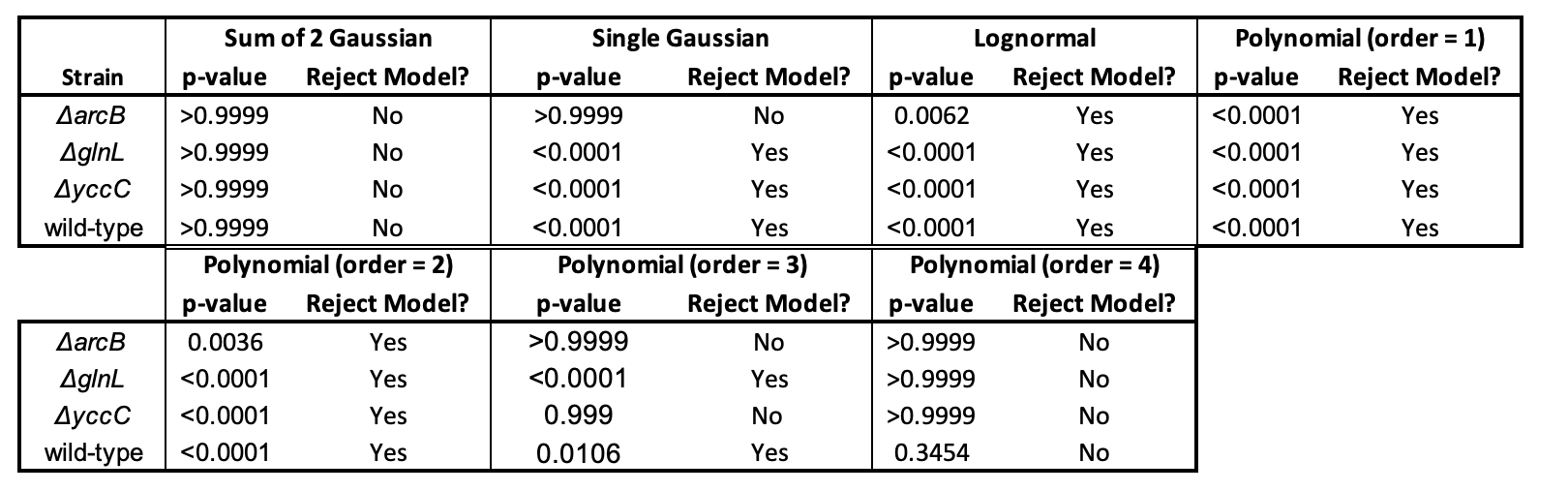
**
