# Supplementary figures and images for: Microbial thermogenesis is dependent on ATP concentrations and the protein kinases ArcB, GlnL, and YccC
Source: PLoS Biol. 2023 Oct 20;21(10):e3002180. doi: 10.1371/journal.pbio.3002180 (PMC10619766; doi:10.1371/journal.pbio.3002180)

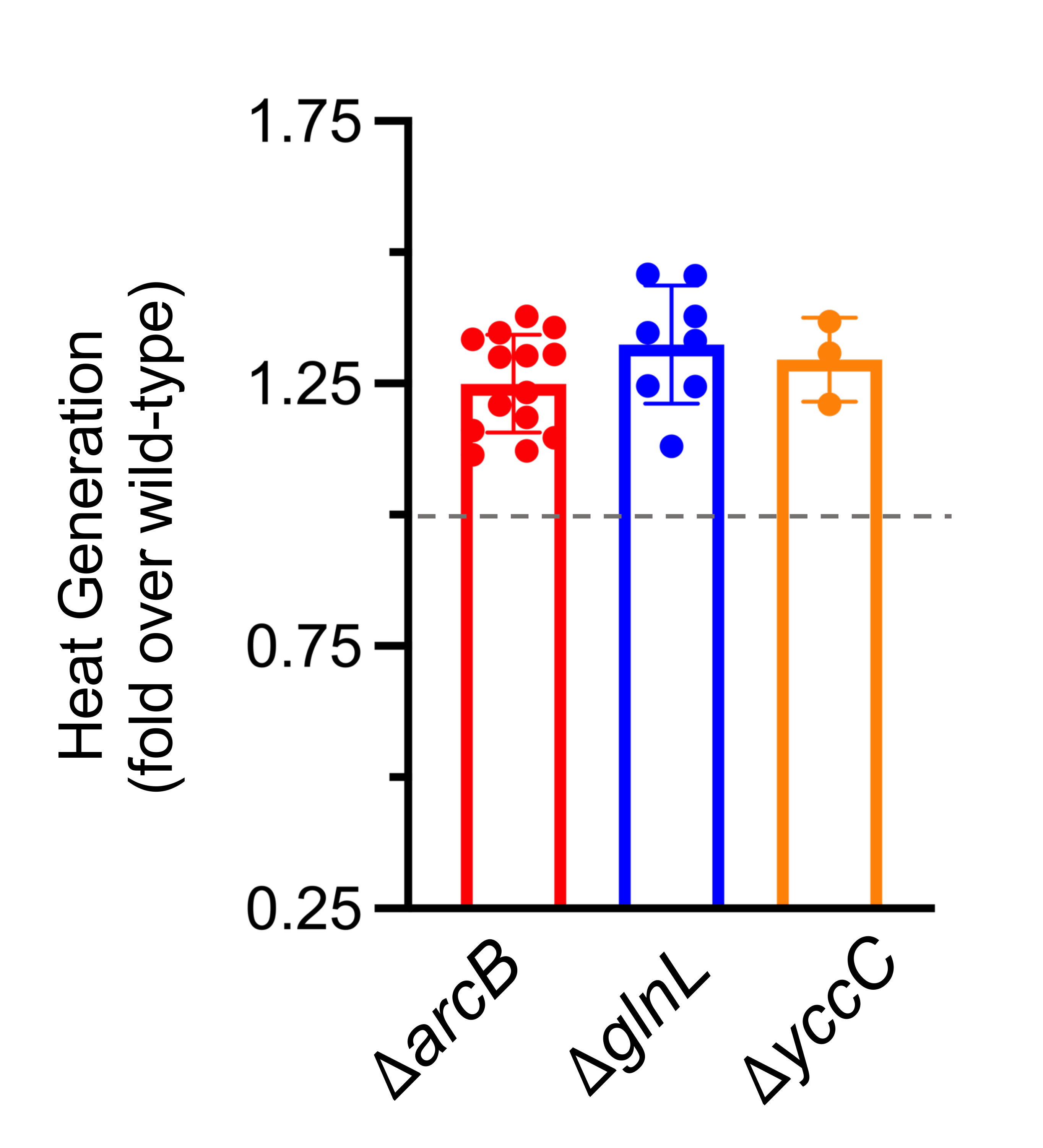

Supplement: S1 Fig — Bar plot of heat generation fold change normalized respective to the experimental wild-type value for high-heat generating strains. Individual experimental values are shown as points. ΔarcB–red, ΔglnL–blue, ΔyccC–orange, and wild-type–the dashed line. Data for all individual replicates can be found in S1 Data. (TIF) [file pbio.3002180.s008.tif]

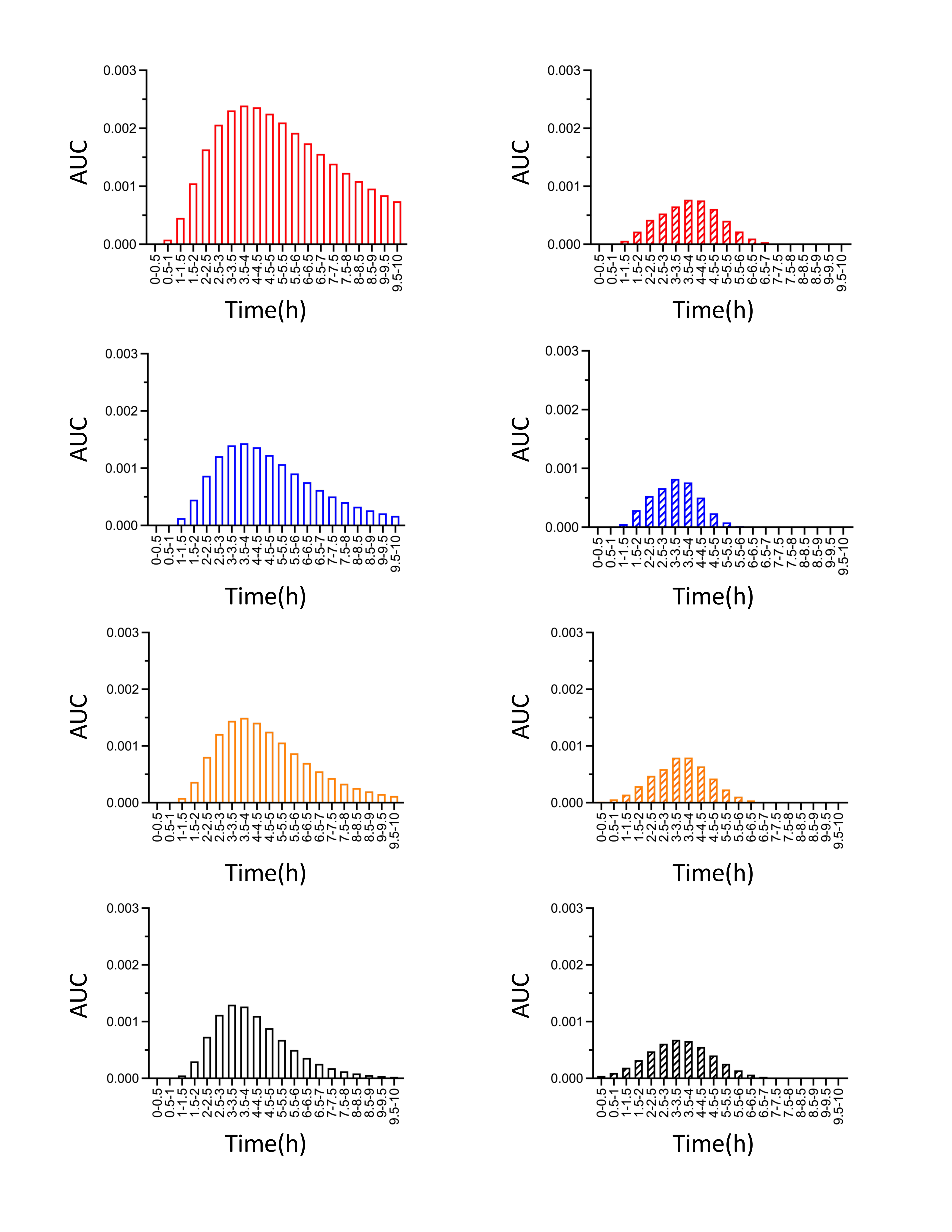

Supplement: S2 Fig — Area under the curve (AUC) analysis was conducted on both intracellular ATP concentration (empty bars) and heat generation curves (striped bars). ΔarcB–red, ΔglnL–blue, ΔyccC–orange, and wild-type–black. Data for all individual replicates can be found in S1 Data. (TIF) [file pbio.3002180.s009.tif]

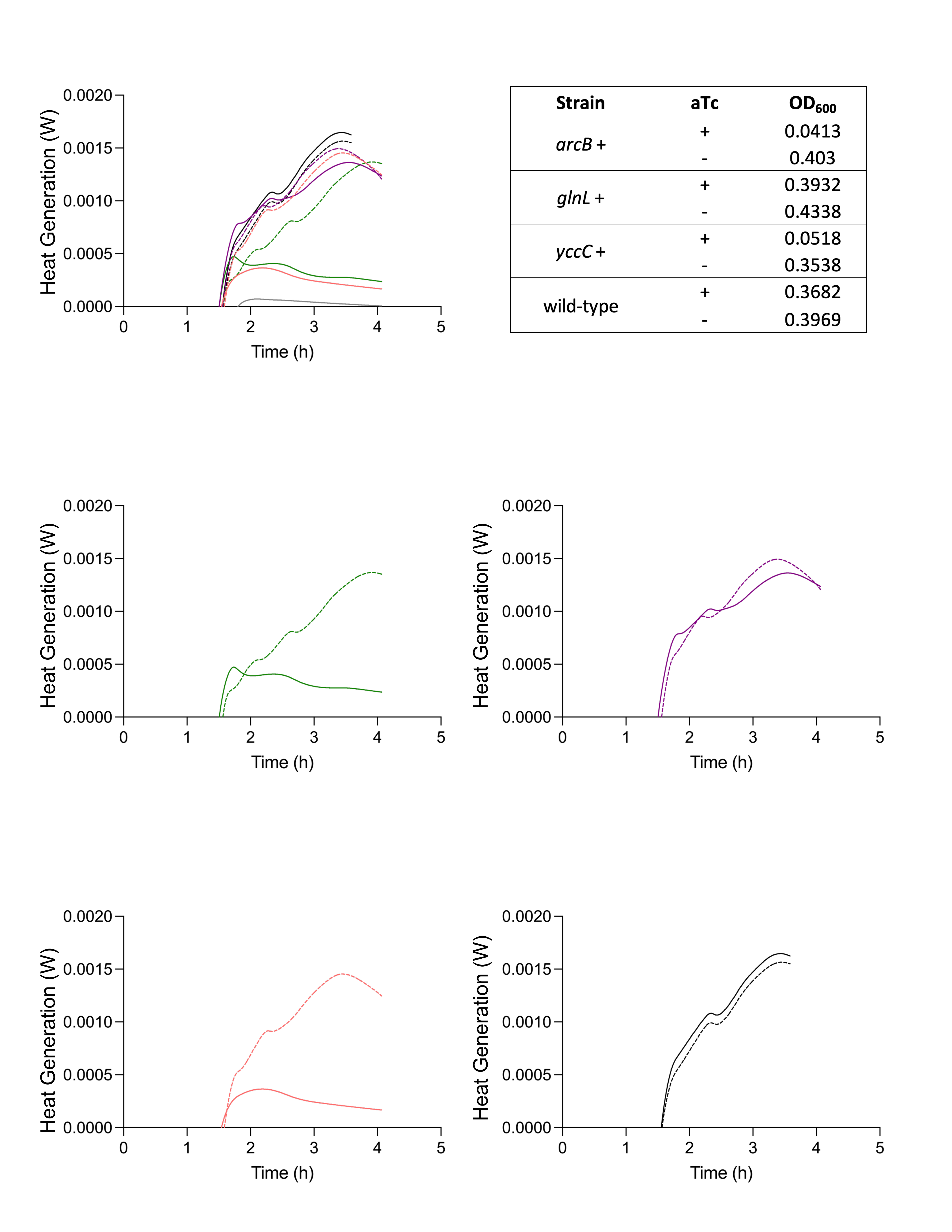

Supplement: S3 Fig — All 3 kinase overexpression strains were evaluated for heat generation (W) over time (h) with and without inducer aTc. Strains grown in the presence of the inducer are shown in solid lines, and uninduced samples are shown in dashed lines. ArcB overexpression (green), GlnL overexpression (purple), YccC overexpression (pink), WT (black), and blank medium negative control (gray). The OD600 values are also shown to note the cell growth at the 4 h time point. Data for all individual replicates can be found in S1 Data. (TIF) [file pbio.3002180.s010.tif]

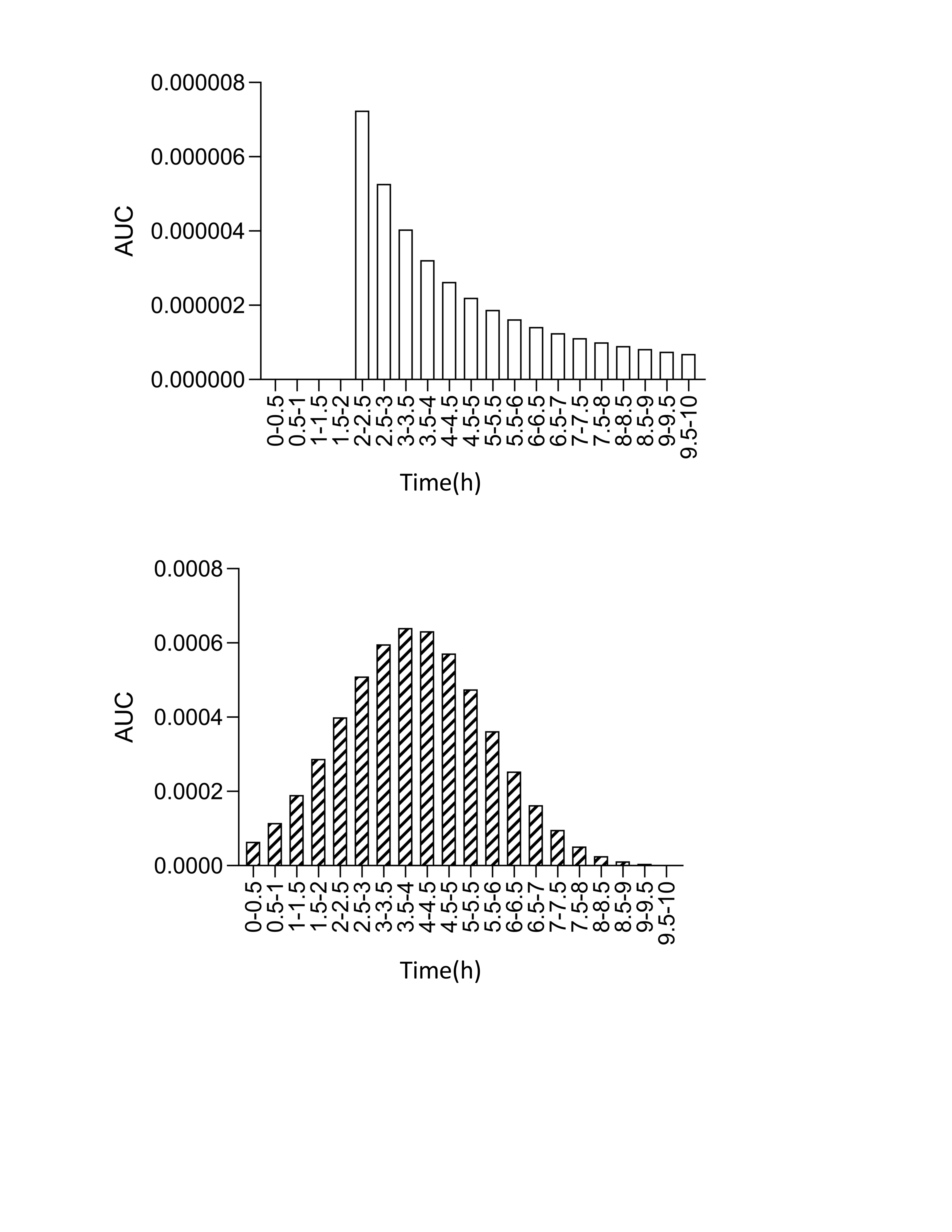

Supplement: S4 Fig — Area under the curve (AUC) analysis was conducted on both intracellular ATP (empty bars) and heat generation curves (striped bars). AUC was conducted by integrating continuous models of the data with a bin size of 0.5 h as the lowest resolution of the measurement. Data for all individual replicates can be found in S1 Data. (TIF) [file pbio.3002180.s011.tif]

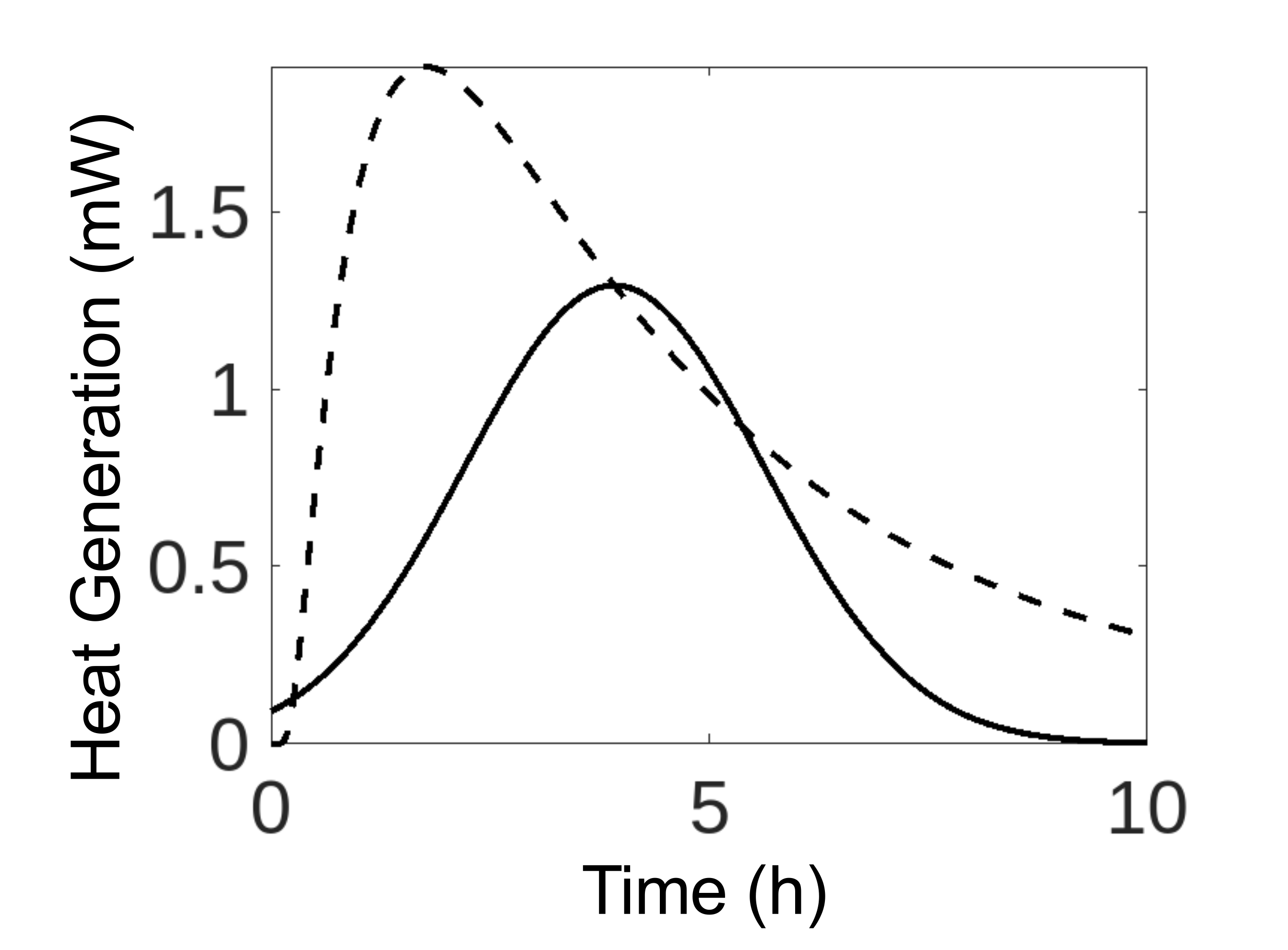

Supplement: S5 Fig — Average heat generation is fitted by the sum of 2 Gaussian equations (solid line) and plotted against the respective MTM approximations for the strain’s heat generation over time (dashed line). Data for all individual replicates can be found in S1 Data. (TIF) [file pbio.3002180.s012.tif]

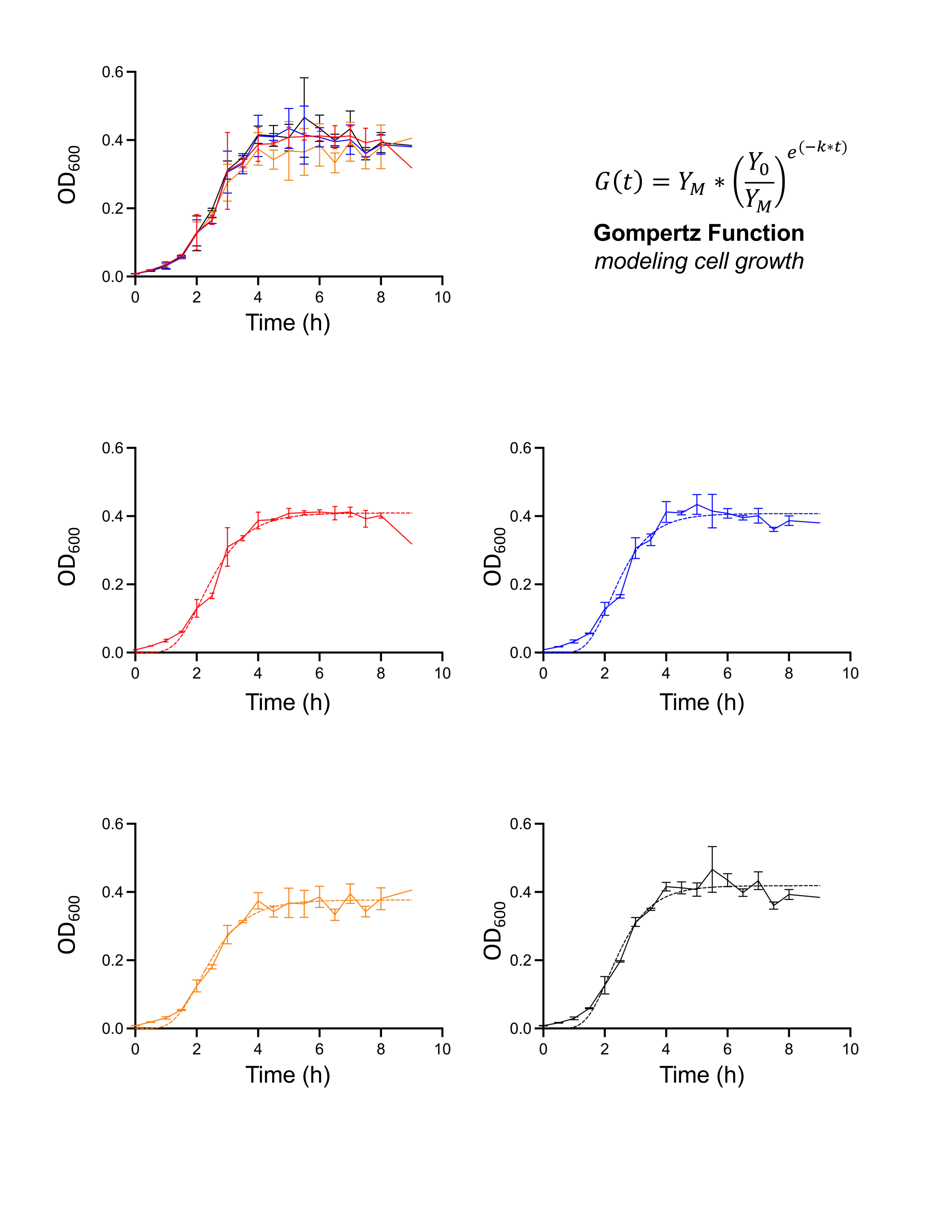

Supplement: S6 Fig — Experimental microaerobic growth data for all strains (OD600—solid line) fit with the Gompertz growth model equation (dashed). ΔarcB–red, ΔglnL–blue, ΔyccC–orange, and wild-type–black. Gompertz growth equation: a–asymptote at t→∞, b–displacement along x-axis, c–growth rate, and t–time. Error bars: ± standard deviation. N = 4 for all strains. Data for all individual replicates can be found in S1 Data. (TIF) [file pbio.3002180.s013.tif]

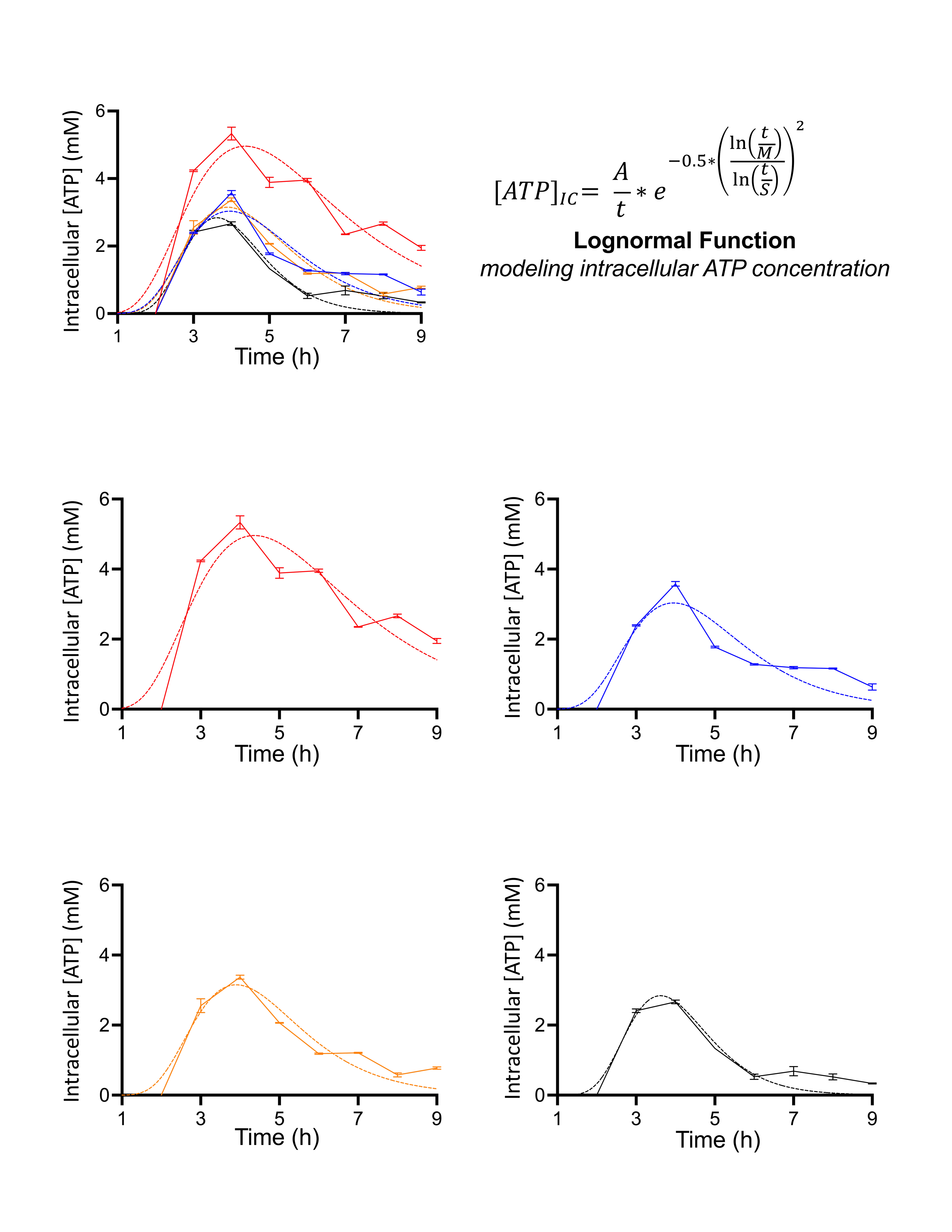

Supplement: S7 Fig — Experimental intracellular ATP concentration data (mM—solid line) fit with the lognormal model equation (dashed). N = 2 for all samples. The model equation for the lognormal function is shown. ΔarcB–red, ΔglnL–blue, ΔyccC–orange, and wild-type–black. Lognormal equation: M–geometric mean, S–geometric standard deviation, t–time, and A–factor related to amplitude. Error bars: ± SD. Data for all individual replicates can be found in S1 Data. (TIF) [file pbio.3002180.s014.tif]

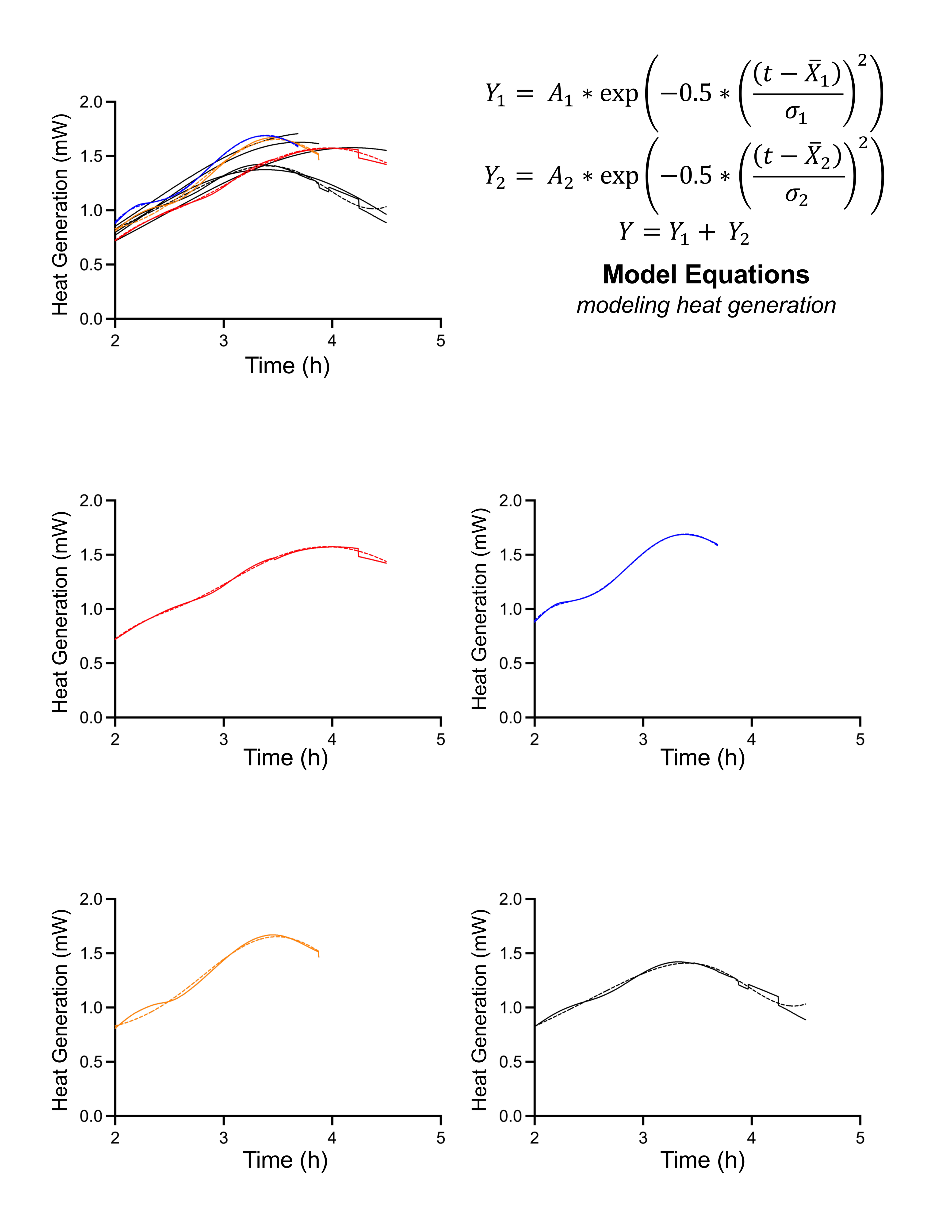

Supplement: S8 Fig — Experimental thermogenic strain heat flow data (mW—solid line) fit with the 2 Gaussian equations (dashed). The model equation for the sum of 2 Gaussian functions is shown. ΔarcB–red, ΔglnL–blue, ΔyccC–orange, and wild-type–black. Sum of 2 (i = 1,2) Gaussian equations: A–amplitude, X¯i–mean, t–time, and σi–standard deviation. Data for all individual replicates can be found in S1 Data. (TIF) [file pbio.3002180.s015.tif]

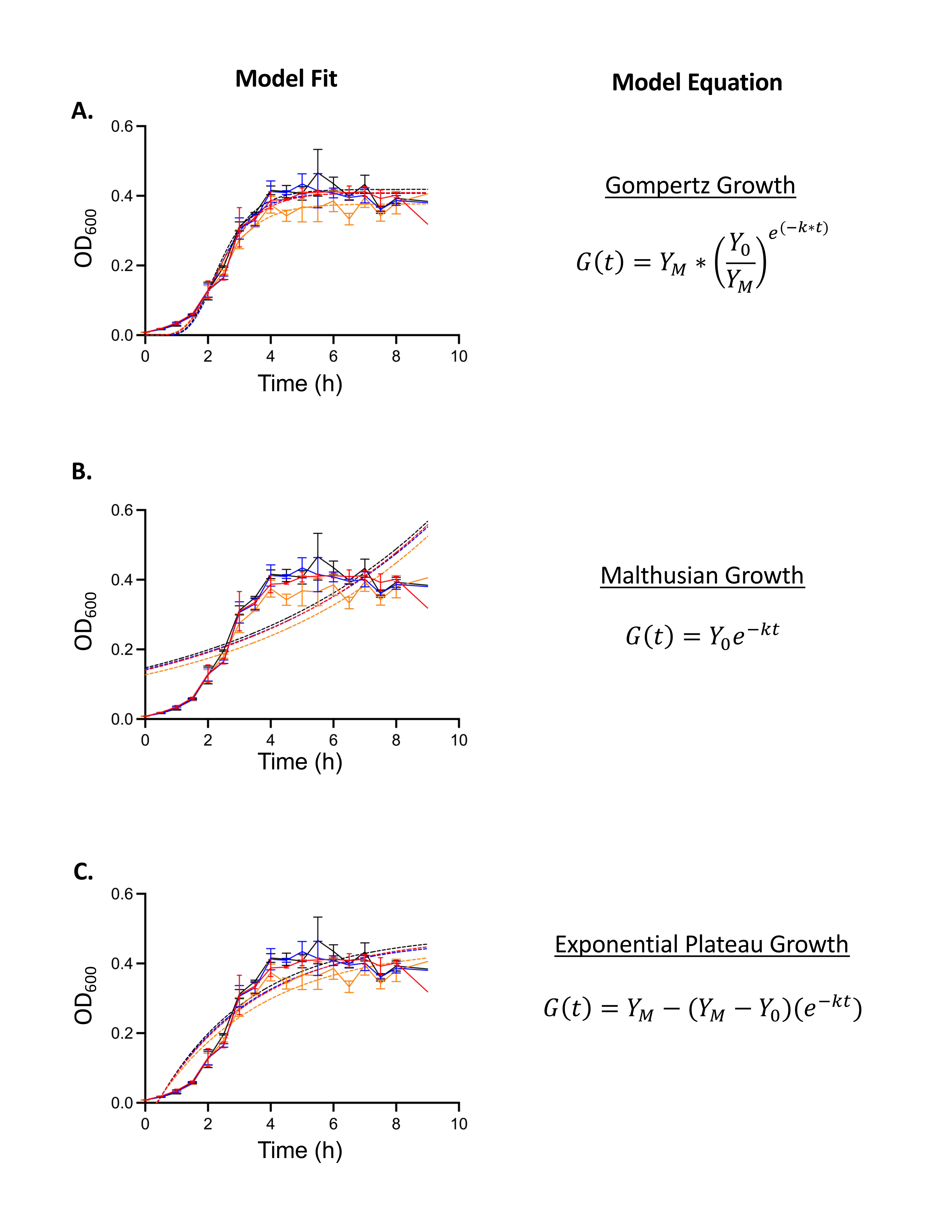

Supplement: S9 Fig — Various regression model equations (dashed line) were fit to the experimental data for microbial growth–G (OD600 –solid line). (A) Gompertz Model: YM—Maximum population, Y0—initial population, k–rate constant, and t–time. (B) Malthusian Model: Y0—initial population, and k–rate constant. (C) Exponential Plateau: YM—Maximum population, Y0—initial population, k–rate constant, and t–time. ΔarcB–red, ΔglnL–blue, ΔyccC–orange, and wild-type–black. Error bars: ± SD. Data for all individual replicates can be found in S1 Data. (TIF) [file pbio.3002180.s016.tif]

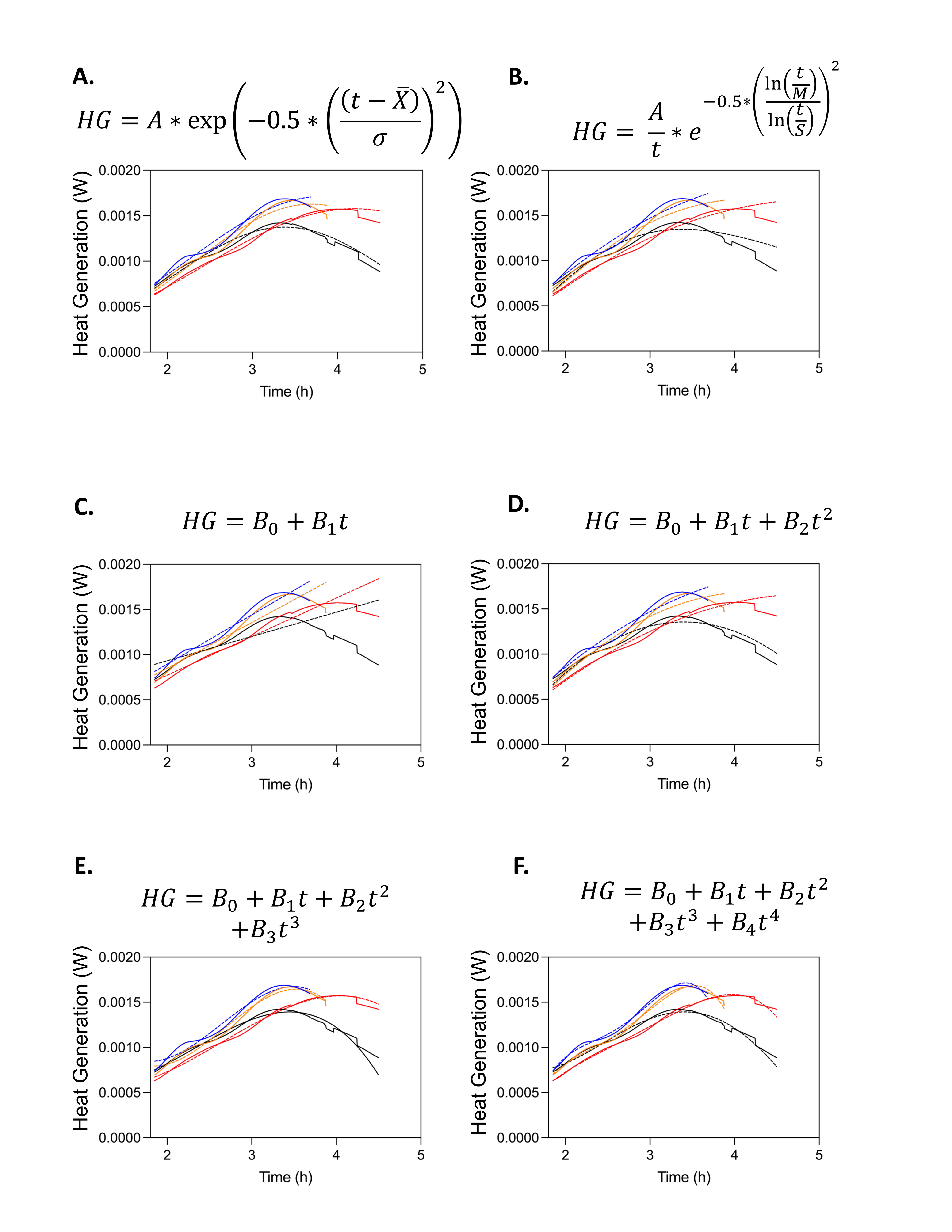

Supplement: S10 Fig — Various regression model equations (dashed line) were fit to the experimental data (W–solid line) for microbial heat generation (HG). (A) Single Gaussian Model: A–amplitude, X¯–mean, t–time, and σ– standard deviation. (B) Lognormal Model: M–geometric mean, S–geometric standard deviation, t–time, and A–factor related to amplitude. (C) First order polynomial: Bi − coefficient, and t–time. (D) Second order polynomial: Bi − coefficient, and t–time, (E) Third order polynomial: Bi − coefficient, and t–time, (F) Fourth order polynomial: Bi − coefficient, and t–time. ΔarcB–red, ΔglnL–blue, ΔyccC–orange, and wild-type–black. Data for all individual replicates can be found in S1 Data. (TIF) [file pbio.3002180.s017.tif]

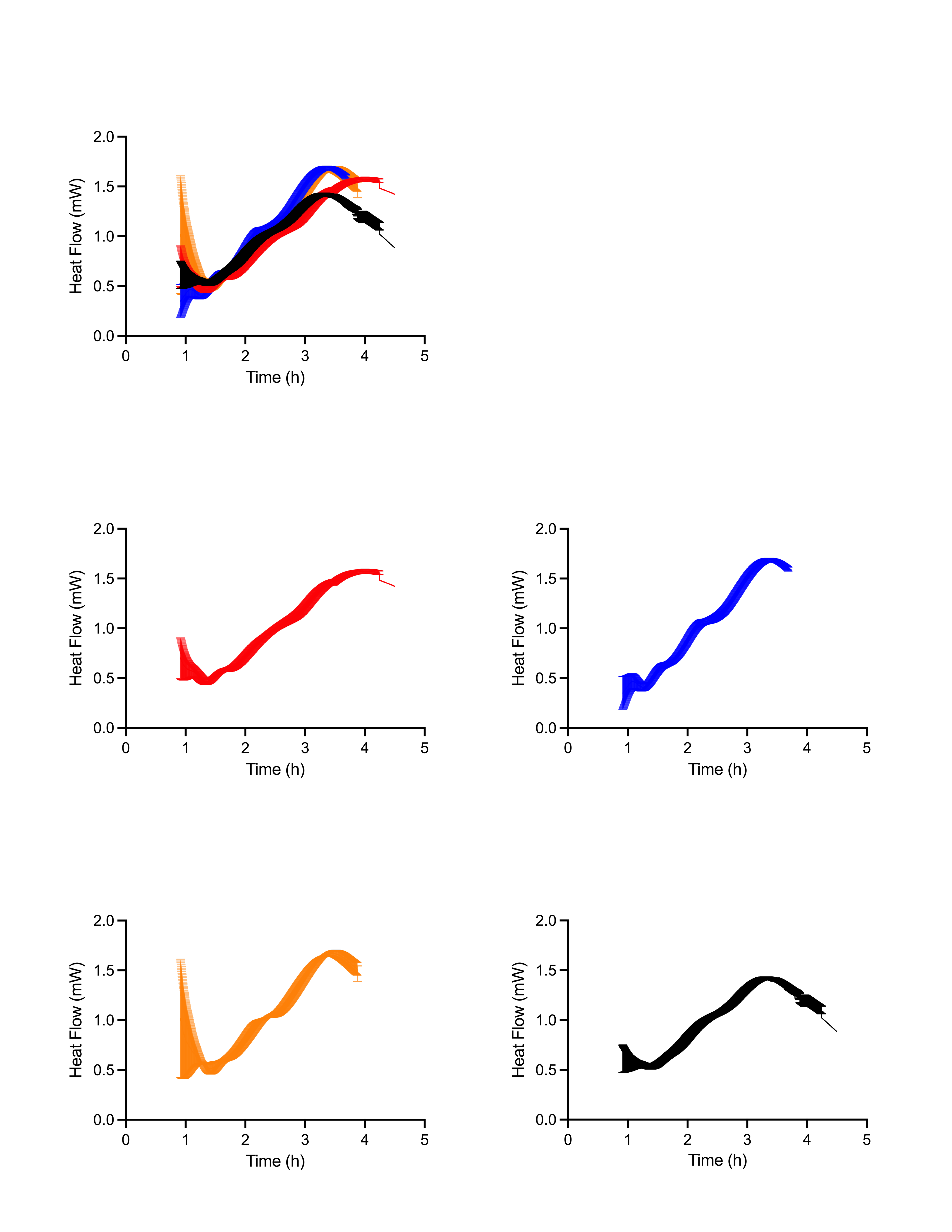

Supplement: S11 Fig — Average strain heat flow (mW) curves over time. All samples were tested in at least triplicate, with distinct samples (N > 3). ΔarcB–red, ΔglnL–blue, ΔyccC–orange, and wild-type–black. Error bars: ± SD. Data for all individual replicates can be found in S1 Data. (TIF) [file pbio.3002180.s018.tif]

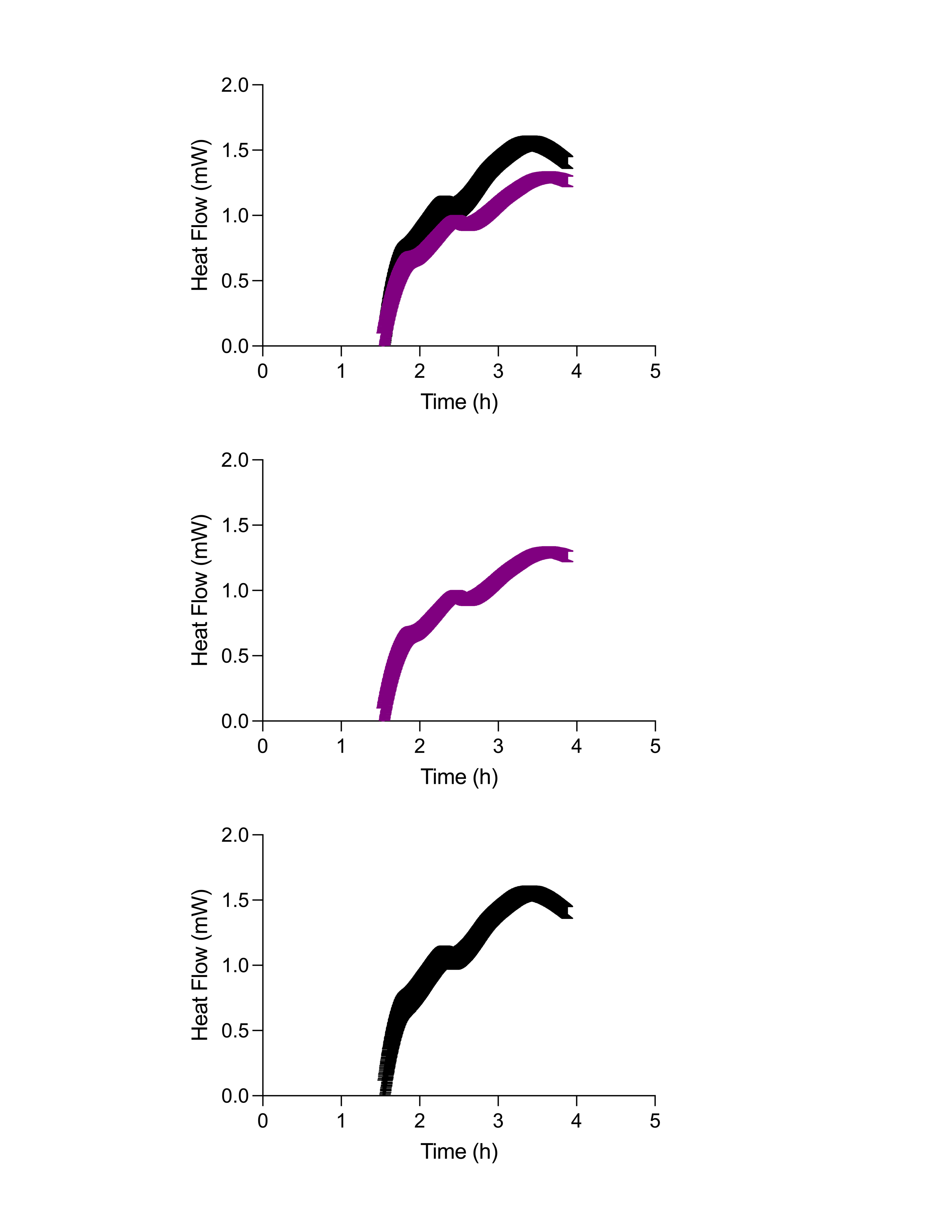

Supplement: S12 Fig — Average strain heat flow (mW) curves over time. All samples were tested in triplicate, with distinct samples. glnL+ (N = 3, purple) and wild-type (N = 3, black). Error bars: ± SD. Data for all individual replicates can be found in S1 Data. (TIF) [file pbio.3002180.s019.tif]
