# Supplementary material for: Microbial thermogenesis is dependent on ATP concentrations and the protein kinases ArcB, GlnL, and YccC
Source: PLoS Biol. 2023 Oct 20;21(10):e3002180. doi: 10.1371/journal.pbio.3002180 (PMC10619766; doi:10.1371/journal.pbio.3002180)
Supplement: Attachment — Submitted filename: Heat_Response_to_Reviewers.docx [file pbio.3002180.s020.docx]

**Response to Reviewers’ Comments**

**PBIOLOGY-D-23-01225R1

Reviewer #1**: Soon and colleagues are describing some mechanisms for heat generation within microbes.
Through a knock out screen, three self-phosphorylating protein kinases were identified to provide extreme thermogenic properties.

This presents a very interesting scientific result which may lead to very interesting applications.

Thanks for the positive comment.

**#major comments**

An expanded discussion of the potential mechanisms integrating the results of the genetic screen, experimental data and simulations.

Thanks for the insightful comment. To address this concern, the following paragraph was added.

“We propose that the putative mechanism that captures the coupled effects of cellular thermogenesis and ATP levels works through the ATP synthase complex. It has recently been recognized that the ATP synthase complex can act in reverse to hydrolyze cellular excesses of ATP in response to increased cellular ATP levels (59). Thus, in the knockout strains presented, where we have an increase in cellular ATP concentrations, this reverse ATP synthase activity can capture the observed coupling of the peaks in the intracellular ATP level and microbial thermogenesis. This mechanism also explains the results of the *glnL* overexpression assay, as a decrease in heat generation is observed with a concomitant reduction in the intracellular ATP level (Figure 4A-D). This type of mechanism, in which the cell balances increased intracellular concentrations of ATP by reversal of ATP synthase activity, follows our kinetic intuition about cellular metabolism. An increase in intracellular ATP is assumed to increase the equilibrium driving force for all reactions involving ATP. This represents a vast and diverse set of enzyme-catalyzed metabolic reactions, with one key similarity – this class of enzymes conserves a common metal cofactor, Mg^2+^. The Mg^2+^ limitation has already been shown to decrease the growth rates of microbial cells such as *E. coli* as this metal cofactor is involved in a wide variety of key cellular processes, including the stabilization of the cellular membrane(60). Thus, the reverse activity of the ATP synthase complex allows the cell to respond to unusually high levels of ATP by hydrolyzing the intracellular excess ATP, to free Mg^2+^ necessary for other key cellular processes.”

Fig.3. It would be very informative to display the variability on the times series of growth, intracellular ATP and heat generation

Thank you for your helpful comment. Figure 3 was edited to include standard error bars for panels A and B. Adding error bars to panel C makes it unreadable (the frequency on the data is every 10 sec, so there are too many error bars). Instead, the figure with the error bars has been added to the supplementary information as Figure S11. Additionally, the caption for Figure 3 was edited, adding: “Error bars: ± s.d.” Accordingly, Figure S6 and S7 were changed from displaying mean only to mean ± standard deviation. The captions were edited by adding: “Error bars: ± s.d.”

**#minor comments**

Fig. 2 A is not readable. Complete listings could be send to supplementary with activity values.

Thank you for this suggestion. Figure 2A was fully edited for increased readability of the figure. Specifically, the gene names were removed and the three important genes for this study (*arcB, glnL,* and *yccC*) are highlighted with a thicker box to highlight the emergence of three strains. Additionally, the full data is included in the supplemental as a separate file (Table S1 in file DataS1).

Fig. 2B maybe could be expanded to separate the four different families of mutants

Thank you for this valuable feedback. Figure 2B was fully edited by increasing the graph size and adding a new figure selecting for the data of the three important mutant strains. The caption was edited by adding: “*ΔarcB, ΔglnL,* and *ΔyccC* data are visualized separately as well.”

Label in Fig. 4 is a bit misleading. I would not use "validation". I think it is more appropriate to talk about Microbial thermogenesis model analysis.

Thank you for your comment. The discussion of “validation” was edited in the caption title:

Microbial thermogenesis model (MTM) validation 🡪 Microbial thermogenesis model (MTM) analysis

MTM model would benefit from more expanded information. Why lognormal was selected? Why the sum of two lognormal? Was it after comparing testing different types of functions? Correlation coefficient are associated with linear model. I wonder how correlation coefficient is here used to describe the model fit.

Thank you for your astute feedback. Tables S3 and S4 were updated to show the p-value and replicate test decision. The methods section was updated as follows:

“**Model Selection**

Model equations for microbial growth, intracellular ATP concentration, and thermogenesis were chosen as follows. A priority of this modeling was to choose analytic functions of time for these models. For intracellular ATP fitting, the lognormal equation, which is frequently used to model biological metabolites, was utilized(65). Model selections for microbial thermogenesis and growth were based on testing a range of different model types and selecting what has the best fit to the data. Goodness of fit was evaluated by replicate test with α = 0.05 (Tables S3 and S4; Figures S9 and S10). The statistical analyses were conducted in GraphPad Prism 9. Area under the curve (AUC) analysis was conducted by integrating the continuous equations of intracellular ATP level and cellular thermogenesis using MATLAB 2022a.”

Table S2 was updated by removing the R^2^ analysis, since this can be biased for nonlinear models. Instead, a standard error of the estimate (SEE) approach was used. This approach was added to the supplemental methods:

“**MTM Evaluation**

To evaluate MTM performance, a standard error in the estimate (SEE) analysis was conducted. SEE is calculated from the following equation:

$$SEE= \sqrt{\frac{\sum{(y_{observed}-y_{predicted})}^{2}}{n}}$$

where $y_{observed}$ are the measured microbial heat outputs (W), $y_{predicted}$ are the predicted microbial heat outputs (W), and $n$ is the total number of sample data points.”

Additionally, the main text of the manuscript was edited to include discussion of this analysis:

“To evaluate MTM’s performance, the standard error of the estimate (SEE) was calculated for both the sum of two Gaussian (2G) equations fit to the observed data and the MTM’s prediction (Table S2). The ∆*arcB* SEE value for the MTM model compared to the experimentally observed data was 0.051, whereas that of the fitted 2G model for this strain’s experimental data was 0.012. Thus, the MTM model predicts heat generation to similar errors as data specifically fit to experimentally observed data. Such an agreement between MTM and the sum of two Gaussian models supports the argument that ATP is the major, even if not the sole, contributor to cellular microbial thermogenesis.”

**Reviewer #2**: Dhatt and Seok Moon present a screen for molecular determinants of thermogenesis in laboratory strains of *Escherichia coli*. Using a set of knockout strains from the Keio collection, the authors assess heat generation during logarithmic growth and identify 3 protein kinases whose deletions dysregulates thermogenesis. Further, they correlate intracellular ATP levels to heat generation. Altogether, the authors present an interesting dive into the molecular mechanisms that govern heat generation during bacterial growth. I believe the manuscript is suitable to publication with some minor additional text edits, analysis, and experiments:

Thank you for the positive comment.

1. The connection between thermogenesis and the microbiome is quite farfetched, especially given the low abundance of *E. coli* in the human microbiome, the vastly altered metabolic state of cells in the gut vs the lab, and the lack of generalizability of the findings to other microbial taxa that dominate the host-associated microbiomes. I would suggest incorporating an expanded and more nuanced discussion if the authors intend to extend these findings to complex microbial communities.

Thank you for your valuable feedback. We have removed all discussion of the application of this technology in the gut microbiome and have amended the section to state:

“We show this engineering potential for the first time through the overexpression of *glnL*. We envision leveraging the foundational understanding of intracellular ATP concentration’s impact on cellular thermogenesis to design high-heat producing organisms. For example, probiotic creams supplemented with an essential nutrient cocktail for high-heat producing cells can be developed. These types of applications could find particular use in cold-water medicine and diving applications.”

2. The model of how the three identified protein kinases play into thermogenesis could be clearer. Unless I am misunderstanding, deletion of any of the kinases leads to increase levels of intracellular ATP, which is correlated with increased heat generation. Since these proteins are deleted, their enzymatic activity on ATP plays no role in heat generation in the deletion mutants, suggesting that these kinases are rather low heat spenders of ATP. Is the model then that ATP can be acted upon by other cellular processes that are more exothermic than kinase activity? If so, what would these processes be?

Thank you for this insightful question and sorry for the confusion. We believe the reviewer may have a slight misunderstanding here. If a knockout of a kinase leads to the increased intracellular ATP level, we believe it is indicative that this gene has high ATP usage activity. However, we do agree that these enzymes’ activities play no direct role in cellular thermogenesis, rather that the effect of their knockout (increasing the intracellular ATP level) leads to increased cellular thermogenesis. The main body of the text has been amended to add our discussion and to clarify our point.

“We propose that the putative mechanism that captures the coupled effects of cellular thermogenesis and ATP levels works through the ATP synthase complex. It has recently been recognized that the ATP synthase complex can act in reverse to hydrolyze cellular excesses of ATP in response to increased cellular ATP levels (59). Thus, in the knockout strains presented, where we have an increase in cellular ATP concentrations, this reverse ATP synthase activity can capture the observed coupling of the peaks in the intracellular ATP level and microbial thermogenesis. This mechanism also explains the results of the *glnL* overexpression assay, as a decrease in heat generation is observed with a concomitant reduction in the intracellular ATP level (Figure 4A-D). This type of mechanism, in which the cell balances increased intracellular concentrations of ATP by reversal of ATP synthase activity, follows our kinetic intuition about cellular metabolism. An increase in intracellular ATP is assumed to increase the equilibrium driving force for all reactions involving ATP. This represents a vast and diverse set of enzyme-catalyzed metabolic reactions, with one key similarity – this class of enzymes conserves a common metal cofactor, Mg^2+^. The Mg^2+^ limitation has already been shown to decrease the growth rates of microbial cells such as *E. coli* as this metal cofactor is involved in a wide variety of key cellular processes, including the stabilization of the cellular membrane(60). Thus, the reverse activity of the ATP synthase complex allows the cell to respond to unusually high levels of ATP by hydrolyzing the intracellular excess ATP, to free Mg^2+^ necessary for other key cellular processes.”

3. The link between ATP hydrolysis could be strengthen by testing conditions wherein intracellular levels of ATP are lowered with respect to wild-type bacteria. Would overexpression of these protein kinases deplete ATP and perhaps lower heat generation? Are there other means to engineer a system with lower ATP concentrations to examine the effect on heat generation?

Thank you for this astute question. To address this excellent point, all three kinases (*arcB*, *glnL,* and *yccC*) were inserted into plasmids for overexpression under an aTc-inducible promoter (P_Tet_). The plasmids were transformed into wild-type *E. coli* BW25113 strains, and the intracellular ATP, growth, and microbial thermogenesis assays were conducted on these strains. The results are tabulated in the new Figures 4, S3, S4, and S5. The manuscript was also edited by adding the following paragraph to the discussion section:

“To evaluate MTM’s performance, the standard error of the estimate (SEE) was calculated for both the sum of two Gaussian (2G) equations fit to the observed data and the MTM’s prediction (Table S2). The ∆*arcB* SEE value for the MTM model compared to the experimentally observed data was 0.051, whereas that of the fitted 2G model for this strain’s experimental data was 0.012. Thus, the MTM model predicts heat generation to similar errors as data specifically fit to experimentally observed data. Such an agreement between MTM and the sum of two Gaussian models supports the argument that ATP is the major, even if not the sole, contributor to cellular microbial thermogenesis.”

Furthermore, the MTM model was applied to the *glnL* overexpression model:

“Finally, the MTM was applied to predict the heat generation in the *glnL*+ strain. The MTM showed a better agreement with knockout strains’ heat generation than that of the *glnL* overexpression strain (Figure S5, Table S2). This disagreement is likely due to the limitations of the ATP assay measurements near or below detectable limits. Since the *glnL* overexpression strain has an increased lag phase, this imprecision becomes more important than the knockout or wild-type strains. This is because the *glnL* overexpression strain peaks in thermogenesis during the early log phase, whereas the kinase knockout and wild type strains peak in late log phase, meaning that they get one clear reading of intracellular ATP before the peak, whereas the *glnL* overexpression strain may not.”

Additionally, the methods section was edited by adding the method of genetic cloning and statement of availability for the plasmid sequences. This necessitated a new citation to help detail the cloning protocol (Rottinghaus et al., 2022).

“**Plasmid construction for kinase overexpression**

Gene coding sequences for *yccC, glnL,* and *arcB* (genome) were amplified from the genomic DNA of *E. coli* BW25113 strain by PCR. All gene sequences were from NCBI genome CP009685.1. The overexpression plasmid backbone was amplified and cloned by Gibson assembly as previously described(62). All plasmid sequences were verified by DNA sequencing (GENEWIZ, South Plainfield, NJ). All the oligonucleotides were purchased from Integrated DNA Technologies (IDT, Coralville, IA). All plasmid minipreps were performed with PureLink™ Quick Plasmid Miniprep Kit (Invitrogen, Waltham, MA). Once plasmid sequences were verified, they were transformed into *E. coli* BW25113 strain and induced with 0.4 ng/mL anhydrotetracycline (aTc), and the resultant strains were assayed for cellular thermogenesis.”

4. In Figure 3, the correlation between ATP and heat is made purely by eye. Could the authors include a more quantitative comparison, perhaps by correlating the AUC of ATP to heat generation in a scatter plot?

Thank you for sharing this great comment. To address this concern, Figures S2 and S4 were added to the supplementary information. These figures detail the recommended AUC analysis, with integrated values combined for each 0.5h time range and plotted in respective time bins on a bar graph. The integration was done using MATLAB.

Additionally, the main text was edited to add discussion of these results:
“The qualitative peak coincidence was confirmed quantitatively by area under the curve (AUC): the maximum integrals of the experimental results’ curves were found between hours 3 and 4 of cell growth (Figure S2).”

Additionally, the methods section for model selection was updated to state:

“AUC analysis was conducted by integrating the continuous fitted equations of intracellular ATP level and cellular thermogenesis using MATLAB 2022a.”
